# Vax1 rs7078160 polymorphisms and non-syndromic cleft lip: correspondence

**DOI:** 10.1016/j.bjorl.2022.01.002

**Published:** 2022-01-29

**Authors:** Rujittika Mungmunpuntipantip, Viroj Wiwanitkit

**Affiliations:** a Private Academic Consultant, Bangkok, Thailand; b Dr DY Patil University, Pune, India

Dear Editor,

We would like to share ideas on the publication “The risk of nonsyndromic cleft lip with or without cleft palate and Vax1 rs7078160 polymorphisms in southern Han Chinese”.^1^ Wang et al. noted that “rs7078160 polymorphism is a risk factor of non-syndromic cleft lip with or without cleft palate, and Vax1 is strongly associated with non-syndromic cleft lip with or without cleft palate in Southern Chinese Han populations”.^1^ We concur that the present study might support that the Vax1 rs7078160 polymorphisms might relate to nonsyndromic cleft lip. Nevertheless, there are other genetic polymorphisms that might have clinical relationship. The examples of those polymorphisms are ABCA4, IRF6 and MTHFD1 gene polymorphisms.^1^, ^2^, ^3^, ^4^ If there is a concurrent of those mentioned polymorphisms, there might be a confounding effect of the present study by Wang et al. Further studies might be conducted in order to assess possible confounding effect from those genetic polymorphisms.

## Authors’ contributions

Rujittika Mungmunpuntipantip: Substantial contributions to study conception and design; Substantial contributions to acquisition of data; Substantial contributions to analysis and interpretation of data; Drafting the article or revising it critically for important intellectual content; Final approval of the version of the article to be published.

Viroj Wiwanitkit: Substantial contributions to study conception and design; Substantial contributions to acquisition of data; Substantial contributions to analysis and interpretation of data; Drafting the article or revising it critically for important intellectual content; Final approval of the version of the article to be published.

## Conflicts of interest

The authors declare no conflicts of interest.
